# Ozone co-exposure modifies cardiac responses to fine and ultrafine ambient particulate matter in mice: concordance of electrocardiogram and mechanical responses

**DOI:** 10.1186/s12989-014-0054-4

**Published:** 2014-10-16

**Authors:** Nicole Kurhanewicz, Rachel McIntosh-Kastrinsky, Haiyan Tong, Leon Walsh, Aimen K Farraj, Mehdi S Hazari

**Affiliations:** Curriculum in Toxicology, School of Medicine, University of North Carolina, Chapel Hill, NC 27599 USA; Department of Environmental Sciences and Engineering, Gillings School of Global Public Health, University of North Carolina, Chapel Hill, NC 27599 USA; Environmental Public Health Division, National Health and Environmental Effects Research Laboratory, US Environmental Protection Agency, Research Triangle Park, Chapel Hill, NC 27711 USA

**Keywords:** Air pollution, Cardiac function, Ventricular contraction, Electrocardiogram, Arrhythmia, Particulate matter

## Abstract

**Background:**

Studies have shown a relationship between air pollution and increased risk of cardiovascular morbidity and mortality. Due to the complexity of ambient air pollution composition, recent studies have examined the effects of co-exposure, particularly particulate matter (PM) and gas, to determine whether pollutant interactions alter (e.g. synergistically, antagonistically) the health response. This study examines the independent effects of fine (FCAPs) and ultrafine (UFCAPs) concentrated ambient particles on cardiac function, and determine the impact of ozone (O_3_) co-exposure on the response. We hypothesized that UFCAPs would cause greater decrement in mechanical function and electrical dysfunction than FCAPs, and that O_3_ co-exposure would enhance the effects of both particle-types.

**Methods:**

Conscious/unrestrained radiotelemetered mice were exposed once whole-body to either 190 μg/m^3^ FCAPs or 140 μg/m^3^ UFCAPs with/without 0.3 ppm O_3_; separate groups were exposed to either filtered air (FA) or O_3_ alone. Heart rate (HR) and electrocardiogram (ECG) were recorded continuously before, during and after exposure, and cardiac mechanical function was assessed using a Langendorff perfusion preparation 24 hrs post-exposure.

**Results:**

FCAPs alone caused a significant decrease in baseline left ventricular developed pressure (LVDP) and contractility, whereas UFCAPs did not; neither FCAPs nor UFCAPs alone caused any ECG changes. O_3_ co-exposure with FCAPs caused a significant decrease in heart rate variability when compared to FA but also blocked the decrement in cardiac function. On the other hand, O_3_ co-exposure with UFCAPs significantly increased QRS-interval, QTc and non-conducted P-wave arrhythmias, and decreased LVDP, rate of contractility and relaxation when compared to controls.

**Conclusions:**

These data suggest that particle size and gaseous interactions may play a role in cardiac function decrements one day after exposure. Although FCAPs + O_3_ only altered autonomic balance, UFCAPs + O_3_ appeared to be more serious by increasing cardiac arrhythmias and causing mechanical decrements. As such, O_3_ appears to interact differently with FCAPs and UFCAPs, resulting in varied cardiac changes, which suggests that the cardiovascular effects of particle-gas co-exposures are not simply additive or even generalizable. Additionally, the mode of toxicity underlying this effect may be subtle given none of the exposures described here impaired post-ischemia recovery.

**Electronic supplementary material:**

The online version of this article (doi:10.1186/s12989-014-0054-4) contains supplementary material, which is available to authorized users.

## Background

Risk assessments of air pollution health effects have become increasingly challenging given the complexity of present-day air pollution mixtures. Epidemiological studies indicate that fine (PM_2.5_) and ultrafine (PM_0.1_) particulate matter (PM) are the principal instigators of adverse clinical events, particularly those involving the cardiovascular system [1]. However, air pollution is a mixture of not only PM, but also gaseous irritants, vapors, and biological substances; thus when examining the effects of any given pollutant, the influence of other components must be considered. As such, studies need to determine whether the resultant physiological and biochemical effects of multipollutant exposures represent the simple additive effects of the pollutants, their synergism or antagonism. One particularly relevant interaction is that of PM and the ubiquitous gaseous co-pollutant O_3_.

Although studies have examined the effects of sequential exposures, for example, ozone (O_3_) and then PM_2.5_ causes decreased HRV, systolic blood pressure and heart rate (HR) in rats [2], only a few studies have addressed the health effects of simultaneous exposures with distinct pollutants and the effects are still not fully clear. For instance, Brook et al. demonstrated acute arterial vasoconstriction in healthy subjects co-exposed to PM_2.5_ and O_3_ [3], whereas Urch et al. [4] found no significant changes in mean arterial pressure, systolic blood pressure or HR in a similar study population; although constriction was observed with PM_2.5_ alone. Animal studies also indicate that the effect of combining pollutants does not necessarily yield the expected synergistic response, especially in the case of susceptible models. Wagner et al. recently showed that depression of heart rate and blood pressure during PM_2.5_ and O_3_ co-exposure was not as great as either pollutant alone in rats fed a high-fructose diet [5]. The respiratory effects of O_3_ and PM co-exposure are equally conflicting. For example, rats instilled with ozonized DEP had increased inflammatory cells and protein in the lungs [6], whereas mice co-exposed to O_3_ and DEP did not have increased cytotoxicity or inflammation [7]. Instead, in this latter study, co-exposed mice had increased bronchoconstriction, which is a measure of lung function. Similar investigations into the effects of simultaneous exposure on cardiac function have not been widely conducted.

Rodent electrocardiograms (ECG) can provide valuable insight into cardiovascular function in air pollution studies, particularly when pollutant concentrations are low and overt inflammation or toxicity are not observed. ECG is now routinely used in rodents for the detection of disturbances in myocardial impulse formation and conduction, as well as abnormal cardiac rhythm and altered autonomic regulation of the heart. As such, a wide range of cardiac responses demonstrated by controlled human and animal PM exposure studies have provided biological plausibility to the health effects of air pollution [3,8-10]. Some of these are responses observed using ECG and have been shown to be similar in humans and animals [11,12]. For instance, some human subjects exposed to PM have decreased heart rate variability (HRV), which is a predictor of increased risk [13-16], and enhanced arrhythmogenesis [17]. Experiments in animals not only show a similar PM-induced decrease in HRV and increased incidence of arrhythmia [1], but also functional decrements in the heart such as diminished left ventricular developed pressure (LVDP) and decreased contractility [18-20]. On the other hand, few studies, if any, have examined the effects of simultaneous PM and O_3_ exposure on both ECG and mechanical function (e.g. contractility) of the heart.

Thus, the purpose of this study was to determine the effects of concentrated ambient particles (CAPs), with and without O_3_ co-exposure, on cardiac electrical and mechanical function in mice. Previous data suggests that PM size determines the physiological impact with fine PM causing primarily pulmonary effects and ultrafine PM altering cardiac function [21,22]. We hypothesized (1) that inhalation of either fine (FCAPs) or ultrafine CAPs (UFCAPs) would cause cardiac electrical dysfunction, mechanical decrements and arrhythmogenesis in mice; but (2) that UFCAPs, due to its size, would have a greater effect on the heart than FCAPs; and (3) that O_3_ co-exposure would potentiate the response elicited by both particle sizes, respectively.

## Results

### Chamber and exposure characteristics

Table 1 shows the concentration and particle size of CAPs and O_3_, and chamber characteristics for each exposure group. Table 2 indicates the elemental composition of the particulate matter from each of the exposure groups. Other than iron (Fe), FCAPs and UFCAPs particulate matter were of similar composition with the majority of the elemental fraction composed of SO_4_.

**Table 1:** **Chamber and exposure characteristics**

	**Groups**					
	**FA**	**UFCAPs**	**UFCAPs + O**_**3**_	**FCAPs**	**FCAPs + O**_**3**_	**O**_**3**_
Temperature (°C)	22.3 ± 0.1	23.0 ± 0.1	22.6 ± 0.1	22.0 ± 0.1	22.2 ± 0.1	22.5 ± 0.2
Rel. humidity (%)	50.2 ± 0.7	70.5 ± 4.6	56.0 ± 3.6	59.8 ± 3.4	59.0 ± 5.8	52.4 ± 2.2
O_3_ (ppb)	4.0 ± 0.0	25.7 ± 4.7	298.3 ± 0.7	33.1 ± 2.0	300.0 ± 0.4	299.0 ± 1.1
PM Mass (ug/m^3^)	4.9 ± 2.2	138.8 ± 33.1	85.7 ± 6.5	190.9 ± 32.8	211.5 ± 37.3	3.4 ± 1.3
PM Total # (particles/cc)	24.2 ± 1.4	2.1E^5^ ± 5.6E^3^	1.6E^5^ ± 2.0E^3^	1.0E^4^ ± 5.2E^1^	1.1E^4^ ± 1.6E^2^	20.2 ± 4.2
Particle size (um)	-	0.076	0.072	0.246	0.235	-
Geo. Std. Dev.	-	1.67	1.66	1.96	1.67	-
	**PM CARBON**					
TC (μg/m^3^)	3.4 ± 0.2	67.6 ± 6.5(48.7%)	46.4 ± 3.4(54.1%)	53.8 ± 4.4 (28.2%)	47.8 ± 7.3 (22.6%)	4.3 ± 0.4
OC (μg/m^3^)	3.7 ± 0.2	64.5 ± 5.9 (46.5%)	44.6 ± 3.1 (52.0%)	50.3 ± 4.0 (26.3%)	45.7 ± 6.6 (21.6%)	4.6 ± 0.4
EC (μg/m^3^)	**	3.1 ± 0.6(2.2%)	1.8 ± 0.3(2.1%)	3.4 ± 0.5(1.8%)	2.2 ± 0.7(1.0%)	**

Reported values are mean ± SEM for each group over all exposure days.

**below detection limit.

Carbon percentages are by mass.

**Table 2:** **Elemental composition of particulate matter in exposure groups**

**Element(μg/m** ^**3**^ **)**	**Groups**					
	**FA**	**UFCAPs**	**UFCAPs + O**_**3**_	**FCAPs**	**FCAPs + O**_**3**_	**O**_**3**_
Al	---	bdl	bdl	1.8364	0.8428⋄	---
As	---	0.0020	0.0040	0.0044	0.0077	---
Ba	---	0.0070	0.0047	0.0865	0.0480⋄	---
Ca	---	bdl	bdl	1.1741	0.6475	---
Cd	---	0.0005	0.0005	0.0015	0.0010	---
Co	---	0.0002	0.0002	0.0008	0.0006	---
Cr	---	0.0088	0.0198	0.0129	0.0134	---
Cu	---	0.0119	0.0727Δ	0.1550	0.0537⋄	---
Fe	---	0.0723	0.0848	2.1031	1.0555⋄	---
K	---	0.7448	0.4325	2.6025	1.3867	---
Li	---	bdl	bdl	0.0017	0.0013	---
Mg	---	0.0282	0.0254	0.5585	0.6145	---
Mn	---	0.0048	0.0058	0.0604	0.0348	---
Mo	---	0.0014	0.0032	0.0033	0.0036	---
Na	---	0.1491	0.2550	0.8990	3.7144	---
Ni	---	0.0053	0.0348Δ	0.0079	0.0084	---
P	---	bdl	bdl	0.1278	0.1565	---
Pb	---	0.0128	0.0124	0.0373	0.0271	---
SO_4_	---	56.4662	39.8730	39.4038	49.3026	---
Sb	---	0.0035	0.0040	0.0146	0.0108	---
Se	---	bdl	bdl	0.0246	0.0248	---
SiO_2_	---	Bdl	bdl	4.9640	2.8436⋄	---
Sn	---	0.0182	0.0239	0.0151	0.0121	---
Sr	---	0.0016	0.0006	0.0179	0.0101	---
Ti	---	bdl	bdl	0.0691	0.0429	---
V	---	0.0023	0.0052	0.0092	0.0122	---
Zn	---	0.1527	0.0989	0.2171	0.2057	---

bdl - Below Detection Level.

--- Very low PM concentrations, insufficient sample mass for elemental analysis.

Δ Significantly different from UFCAPs.

⋄Significantly different from FCAPs.

### Estimated particle doses

The following particle doses were calculated for the mice in each of the PM-exposed group: (1) UFCAPs - 0.418 μg (2) FCAPs - 0.426 μg (3) UFCAPs + O_3_ - 0.264 μg and (4) FCAPs + O_3_ - 0.446 μg. Using the same model and exposure characteristics the estimated human doses were determined to be: (1) UFCAPs - 103.4 μg (2) FCAPs - 81.3 μg (3) UFCAPs + O_3_ - 65.8 μg and (4) FCAPs + O_3_ - 85.0 μg.

### Heart rate

**Figure 1:**
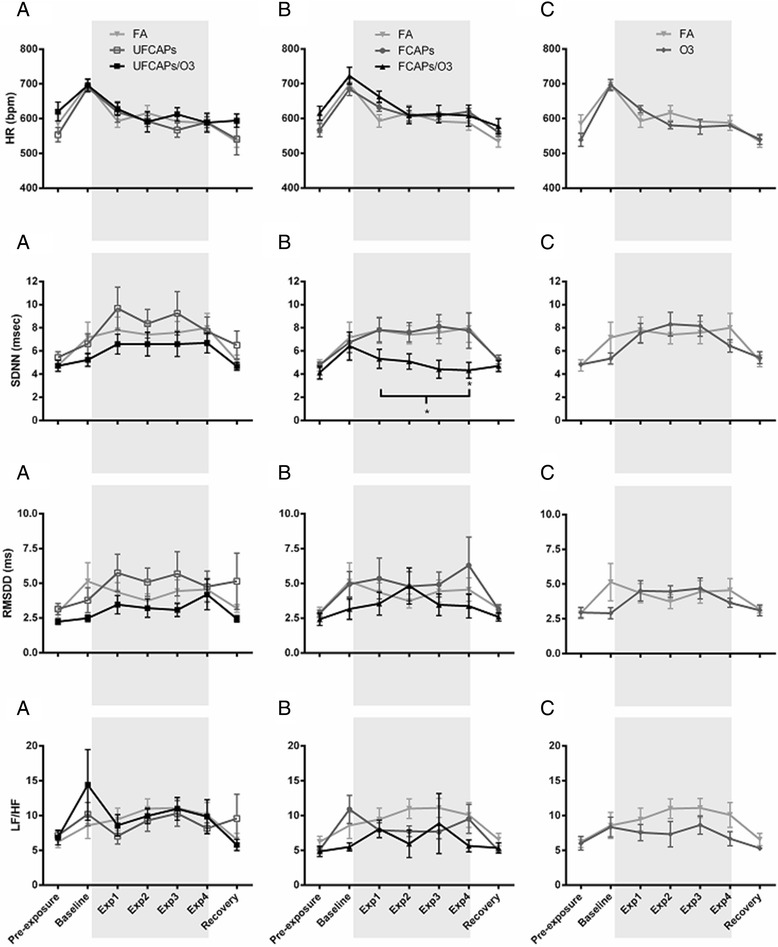
**The effect of CAPs with and without O**_**3**_ **on heart rate and heart rate variability.** All animals were placed in the chambers and allowed to acclimate for 1 hr before the exposure began (Baseline) and then exposed for 4 hrs (shaded area). When compared to pre-exposure, all animals experienced an increase in HR during baseline, and then a progressive decrease from baseline during hour-1, 2, 3 and 4 of exposure (Exp1, Exp2, Exp3, and Exp4). Exposure to UFCAPs with (UF/O_3_) or without (UF) ozone did not cause any significant changes in HR or HRV at any time point (Column **A**.). Similarly, there was no effect of FCAPs alone (F) on any parameter; only exposure to FCAPs + O_3_ (F/O_3_) significantly decreased SDNN when compared to FA (Column **B**.). Exposure to ozone alone did not cause any significant effects (Column **C**.). Bracket indicates that each hour of the exposure period is significantly different. Values are mean ± SEM; *p < 0.05, significantly different from FA (n = 6).

Although all animals experienced an increase in HR while in the exposure chamber before the start of the exposure (Baseline) and a progressive decrease during the 4-hour exposure (Exp1, Exp2, Exp3 and Exp4), there were no significant differences in HR among any of the exposure groups during any time period (Figure 1).

### Heart rate variability (HRV)

Exposure to FCAPs + O_3_ caused a significant decrease in the SDNN (4.8 ± 0.4 ms) compared with FA controls (7.7 ± 0.5 ms) (Figure 1). No other significant differences in time-domain HRV measurements were found among any of the exposure groups pre-, during or post-exposure. There were also no significant differences in the LF/HF between any exposure groups.

### Electrocardiogram

**Figure 2:**
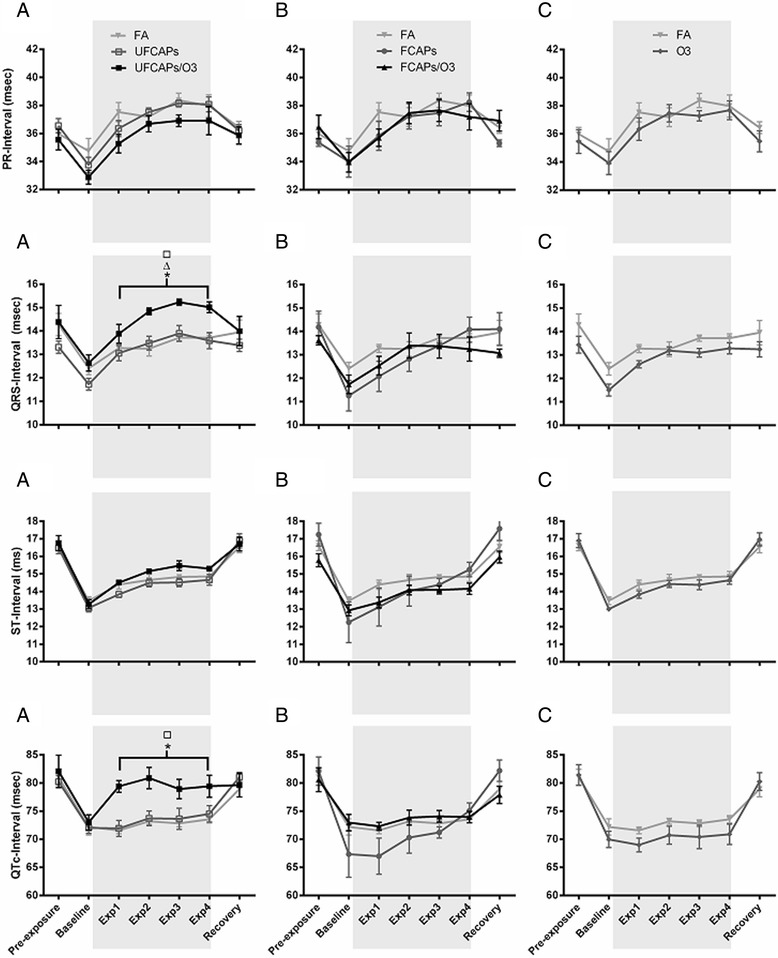
**Electrocardiogram effects before, during and after exposure to CAPs alone or with O**_**3**_ **.** All animals were placed in the chambers and allowed to acclimate for 1 hr before the exposure began (Baseline) and then exposed for 4 hrs (shaded area). All animals experienced a decrease in PR interval, QRS complex duration, ST interval and QTc from pre-exposure to baseline; these changes likely corresponded to the change in HR. Although both PR and ST intervals increased in all groups during hour-1, 2, 3, and 4 of exposure (Exp1, Exp2, Exp3 and Exp4), there were no differences in either of these parameters among any of the groups. QRS also showed an increasing trend during exposure in all groups; however only mice exposed to UFCAPs + O_3_ had a significant increase in QRS and QTc when compared to FA (Column **A**.). There were no significant effects of FCAPs, with or without O_3_, or O_3_ alone at any time points (Column **B**. and **C**., respectively). Bracket indicates that each hour of the exposure period is significantly different. Values are mean ± SEM. p < 0.05; *significantly different from FA, ∆ significantly different from UFCAPs alone, □ significantly different from O_3_ alone (n = 6).

Figure 2 shows the electrocardiogram data before, during and after exposure. There were no significant differences in ECG between any of the groups during pre-exposure or recovery. All animals experienced a decrease in PR interval, QRS, ST interval, and QTc during the baseline, which was likely related to the increase in HR. Thereafter, PR interval and ST interval increased in all animals during the exposure; though there were no significant differences. In contrast, QRS and QTc were significantly increased in mice exposed to UFCAPs + O_3_ when compared to FA. Exposure to O_3_ alone demonstrated a trend towards decreased QTc when compared with FA.

### Cardiac arrhythmia

**Figure 3:**
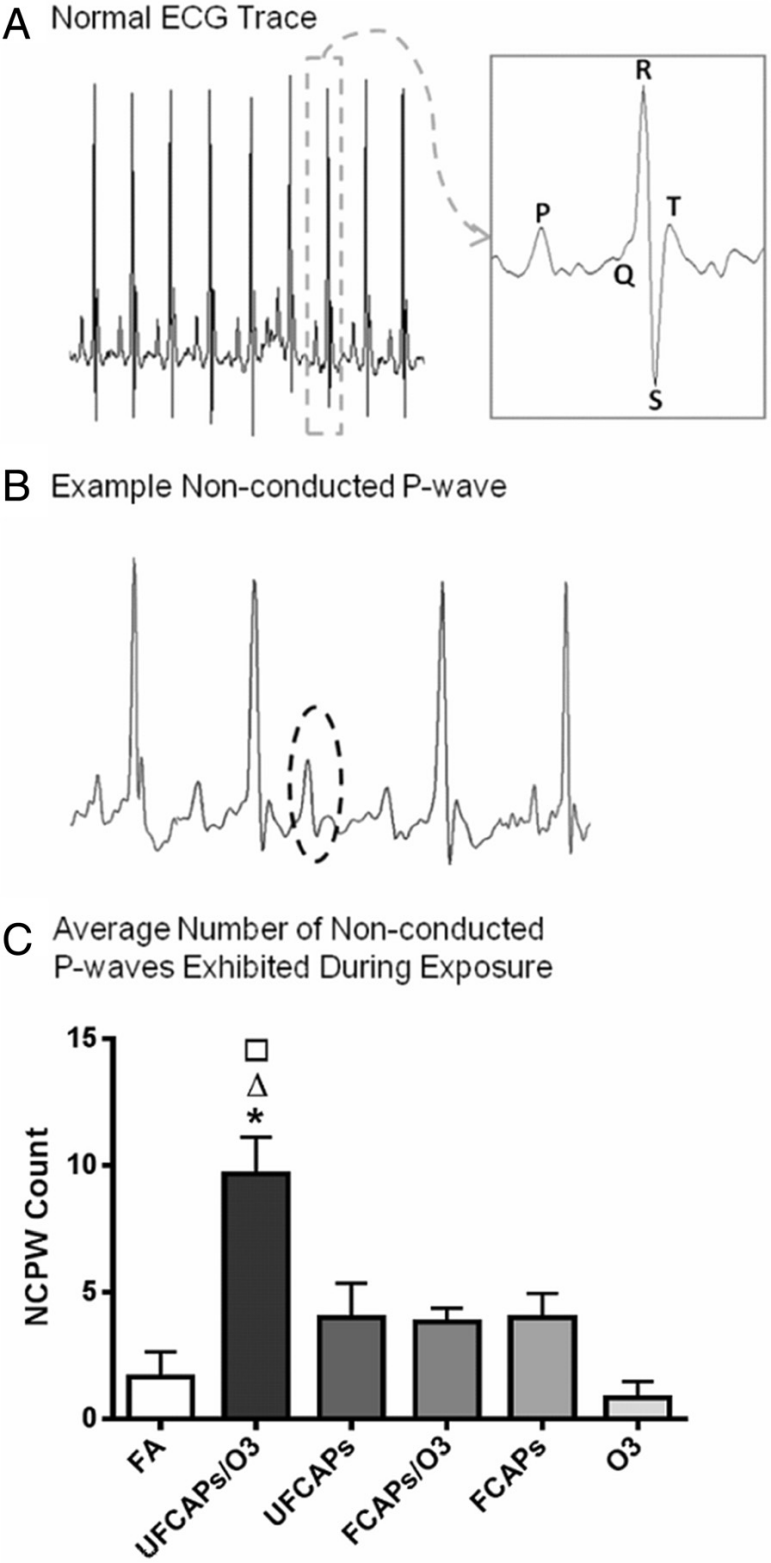
**Typical mouse electrocardiogram and arrhythmia count during exposure. A**. Typical mouse ECG during normal sinus rhythm and **B**. a non-conducted p-wave (NCPW) - represents a sudden loss of conduction from the atria to the ventricles. **C**. Non-conducted p-waves were significantly increased only in mice exposed to UFCAPs + O_3_. Values are mean ± SEM. p < 0.05; *significantly different from FA, ∆ significantly different from UFCAPs alone, □ significantly different from O_3_ alone (n = 6).

There was a significant increase in the number of non-conducted P-wave arrhythmias during the 4-hour exposure period to UFCAPs + O_3_ when compared with FA (Figure 3C). No other significant differences in arrhythmias were observed among any of the exposure groups. Although other types of arrhythmias were noted, they were few in number and not statistically different between any of the groups.

### Cardiac effects before ischemia

**Figure 4:**
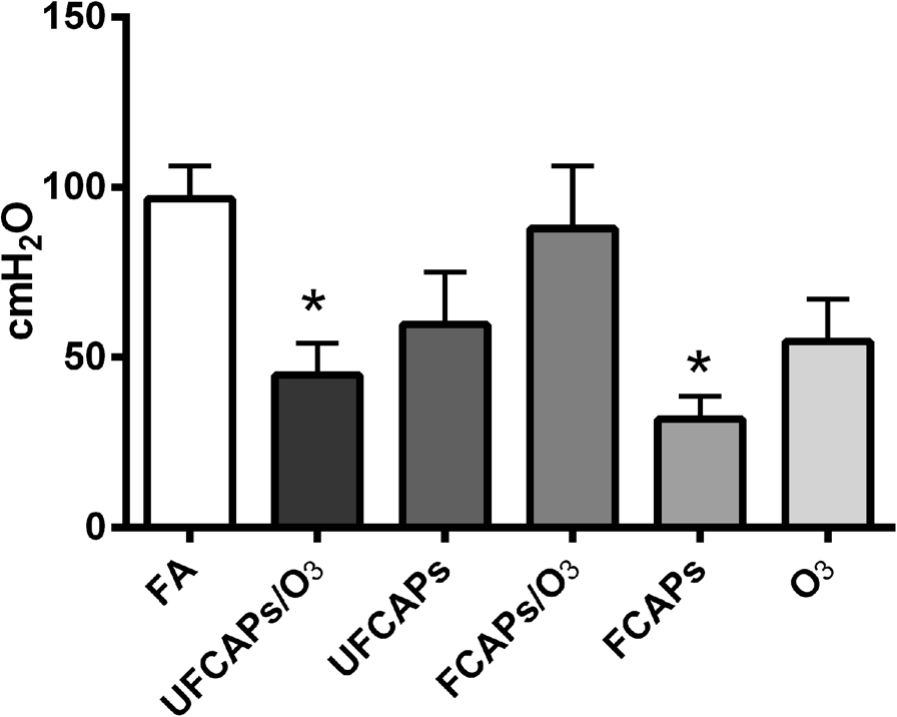
**Effect of CAPs exposure on left ventricular developed pressure (LVDP).** Exposure to FCAPs alone (F) significantly decreased LVDP at baseline (24 hrs after exposure - prior to ischemia) when compared to FA, however there was no effect with O_3_ co-exposure. In contrast, UFCAPs alone had no effect on LVDP but with O_3_ co-exposure caused a significant decrease when compared to FA. Values are means ± SEM (*n* = 5-8/group). *Significantly different from FA; p < 0.05.

**Figure 5:**
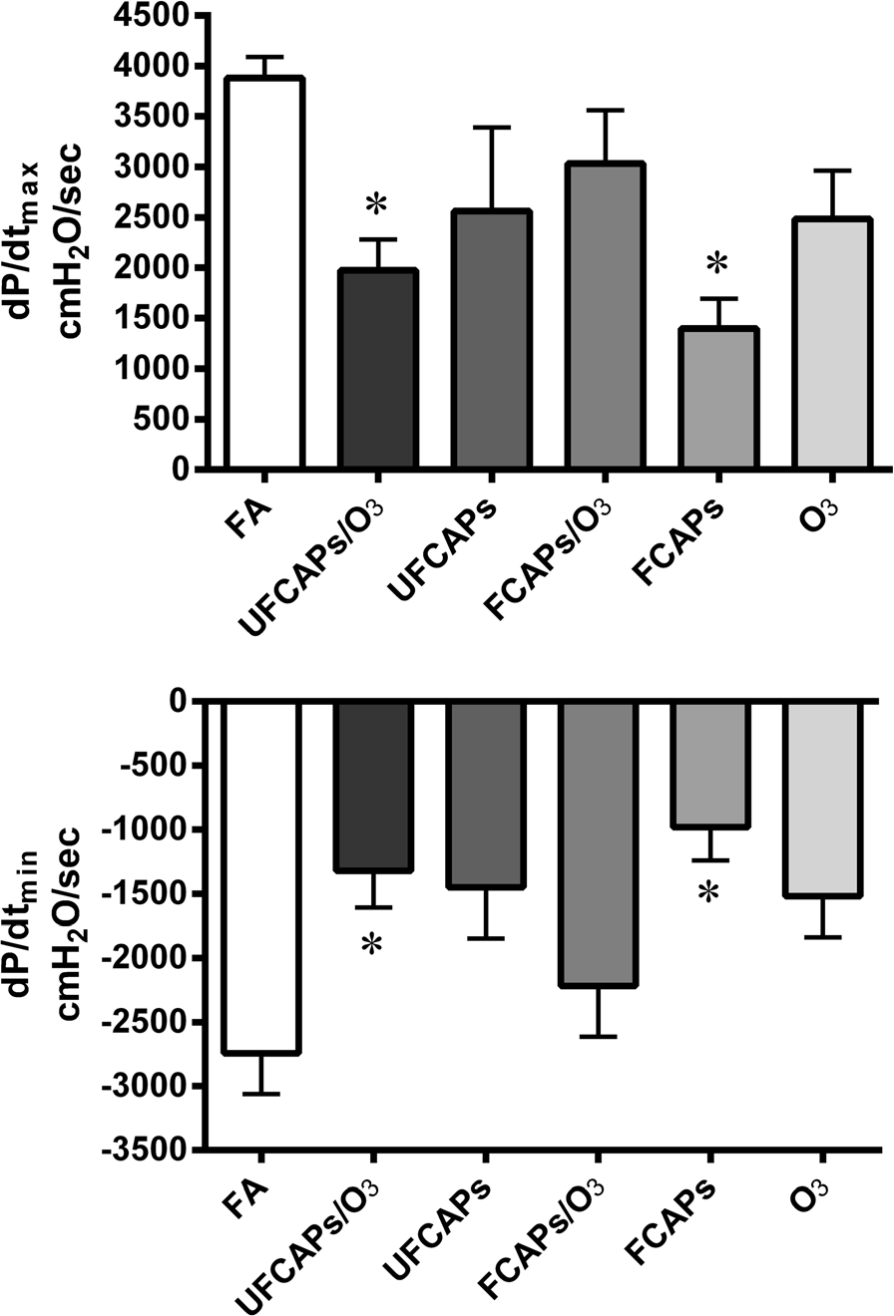
**Effect of CAPs exposure with and without O**_**3**_ **on rate of left ventricle contractility and relaxation.** Assessment of contractility (d*P*/d*t*_max_ - upper panel) and lusitropy (d*P*/d*t*_min_ - lower panel) were carried out at baseline 24 hrs after exposure - prior to ischemia. Exposure to FCAPs alone significantly decreased d*P*/d*t*_max_ and d*P*/d*t*_min_ at baseline when compared to FA, however there was no effect with O_3_ co-exposure (FCAPs/O_3_). In contrast, UFCAPs alone had no effect; but, O_3_ co-exposure with UFCAPs (UFCAPs/O_3_) caused both d*P*/d*t*_max_ and d*P*/d*t*_min_ to significantly decrease when compared to FA. Values are means ± SEM (*n* = 5-8 in each group). *Significantly different from FA; p < 0.05.

Post-exposure (baseline) hemodynamics and the onset time to ischemic contracture for each of the exposure groups are listed in Table 3. As shown in Figure 4, there was a significant decrease in LVDP in the FCAPs (31.9 ± 6.7 cmH_2_O), O_3_ (54.7 ± 12.6 cmH_2_O) and UFCAPs + O_3_ (45.0 ± 9.2 cmH_2_O) groups compared to FA (96.7 ± 9.6 cmH_2_O) 24 hours after exposure and before ischemia. Left ventricular contractility was also significantly depressed in the UFCAPs, FCAPs, O_3_ and UFCAPs + O_3_ groups compared to the FA control group. The maximum d*P*/d*t* was significantly lower in FCAPs (1397 ± 296 cmH_2_O/sec), O_3_ (2483 ± 480 cmH_2_O/sec) and UFCAPs + O_3_ (1975 ± 306 cmH_2_O/sec) when compared to FA (3880 ± 208 cmH_2_O/sec) and the minimum d*P*/d*t* before ischemia was also significantly lower in the UFCAPs (-1452 ± 395 cmH_2_O/sec), FCAPs (-982 ± 259 cmH_2_O/sec), O_3_ (-1520 ± 318 cmH_2_O/sec) and UFCAPs + O_3_ (-1323 ± 286 cmH_2_O/sec) groups when compared to FA (-2744 ± 317 cmH_2_O/sec) (Figure 5; Table 3). There was no difference in HR, coronary flow rate or ischemic contracture between any exposure groups before ischemia (Table 3).

**Table 3:** **Baseline hemodynamic properties and the onset time to ischemic contracture**

**Group (n = 6-8)**	**LVDP (cmH**_**2**_ **O)**	**HR (bpm)**	**Flow rate (mL/min)**	**d** ***P*** **/d** ***t***_**max**_ **(cmH**_**2**_ **O/sec)**	**d** ***P*** **/d** ***t***_**min**_ **(cmH**_**2**_ **O/sec)**	**Time to contracture (min)**
FA	96.7 ± 9.6	306.6 ± 17.5	1.7 ± 0.3	3880 ± 208	-2744 ± 317	14.3 ± 1.9
UFCAPs	59.7 ± 15.4	270.3 ± 19.6	1.7 ± 0.5	2564 ± 825	-1452 ± 395	14.6 ± 1.8
UFCAPs + O_3_	45.0 ± 9.3*	295.1 ± 18.5	2.0 ± 0.5	1975 ± 306*	-1323 ± 286*	15.1 ± 1.6
FCAPs	31.9 ± 6.7*	301.3 ± 27.6	5.7 ± 2.9	1397 ± 296*	-981 ± 259*	11.1 ± 0.5
FCAPs + O_3_	88.0 ± 18.4	277.6 ± 15.7	1.7 ± 0.2	3034 ± 528	-2219 ± 394	12.9 ± 1.3
O_3_	54.7 ± 12.6	246.3 ± 36.4	2.5 ± 0.6	2483 ± 480	-1520 ± 318	15.3 ± 1.6

Values are means ± SEM. Flow rate = coronary flow rate; d*P*/d*t*_max_ = maximum 1st derivative of the change in left ventricular pressure/time; d*P*/d*t*_min_ = minimum 1st derivative of the change in left ventricular pressure/time; time to contracture = onset time to ischemic contracture. *Significantly different from FA; *p* < 0.05; n = 5-8.

Multivariate analysis of variance demonstrated that differences in LVDP, maximum d*P*/d*t* and minimum d*P*/d*t* between the FCAPs alone and FCAPs + O_3_ groups could be accounted for by the decrease in aluminum (Al), barium (Ba), copper (Cu), iron (Fe) or silicon dioxide (SiO_2_) compositions (Table 2); these elements clustered together however the analysis could not determine which element specifically was responsible. There were no apparent differences in elemental composition between UFCAPs alone and UFCAP + O_3_, except nickel (Ni), which were linked to any cardiac response changes, nor were there any other significant linkages with any other cardiac endpoints.

### Cardiac effects post-ischemia

**Figure 6:**
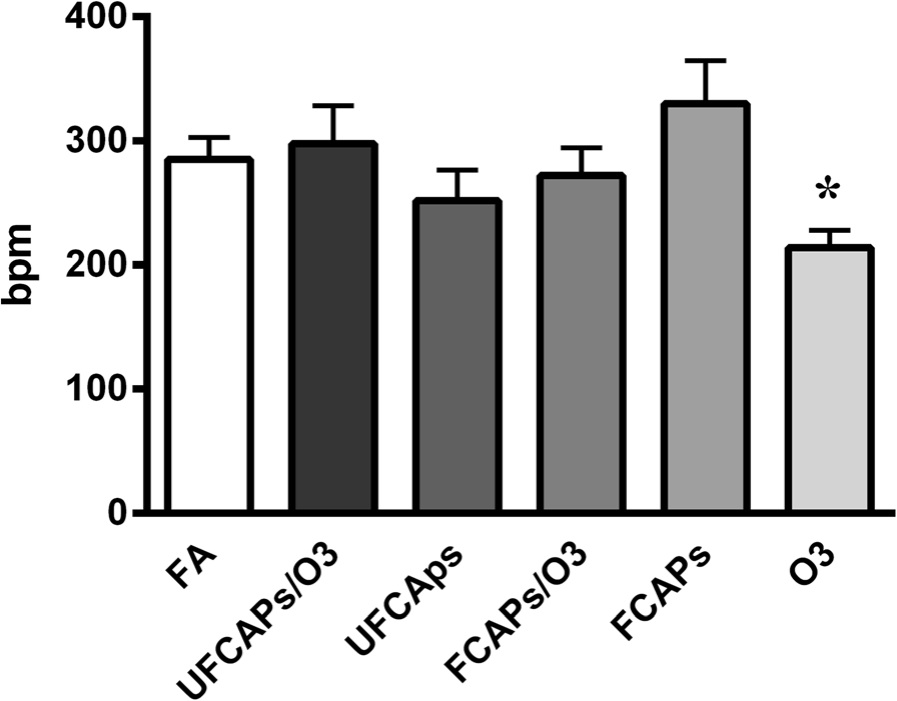
**Heart rate twenty minutes after ischemia-reperfusion.** After 20 mins of reperfusion, heart rate (HR) was significantly lower in animals exposed to O_3_ when compared to FA. There were no other significant differences in post-ischemia HR between any groups or at any other time point. Values are means ± SEM (*n* = 5-8 in each group). *Significantly different from FA; p < 0.05.

**Figure 7:**
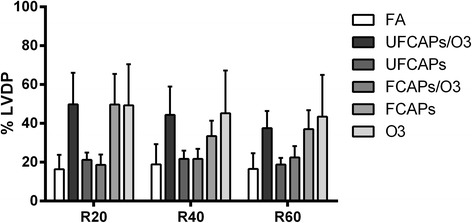
**Post-ischemia recovery of LVDP.** Following ischemia, all animals experienced a significant decrease in recovery LVDP (expressed as a percentage of pre-ischemia) when compared to pre-ischemia. There were no significant differences among any groups in the recovery LVDP at 20 (R20), 40 (R40) or 60 (R60) minutes post-ischemia; however there was a trend towards increased % LVDP in the FCAPs, O_3_ and UFCAPs + O_3_ groups at R20, R40 and R60 when compared to FA; consequently, these groups were the same ones demonstrating pre-ischemia changes. Values are means ± SEM (*n* = 5-8 in each group). *Significantly different from FA; p < 0.05.

After ischemia there were minimal differences among the groups. There was a significant decrease in HR 20 min after reperfusion in the O_3_ group (213.8 ± 14.2 bpm) compared to FA (285.3 ± 17.5 bpm) (Figure 6). There were no differences in the post-ischemia coronary flow rates of any of the groups. Although all groups experienced a significant decrease in LVDP recovery when compared to pre-ischemia, there was no significant difference in post-ischemia recovery of LVDP (Figure 7), d*P*/d*t*_max_, and d*P*/d*t*_min_ between any exposure groups.

### Biochemical markers and inflammatory cells in BAL and blood

Exposure to O_3_ alone or UFCAPs + O_3_ caused a significant decrease in glutathione S-transferase (GTR) when compared to controls. There were no other significant differences in any other BAL cells or markers, or any of the serum or plasma markers (Table 4).

**Table 4:** **Biochemical markers in the bronchoalveolar lavage and serum**

	**Bronchoalveolar lavage**				**Blood**							
**Group**	**LDH(U/L)**	**MIA(μg/ml)**	**NAG(U/L)**	**Protein(μg/ml)**	**ACE(U/L)**	**CK(U/L)**	**CRP(μg/dl)**	**HBDH(U/L)**	**GTR(IU/ml)**	**LDH(U/L)**	**SOD(U/ml)**	**Protein(g/dl)**
Air	67.2 **±** 12.4	10.3 **±** 0.4	6.0 **±** 0.2	131.0 **±** 9.5	193.9 **±** 26.4	2.3E3 **±** 891.5	64.8 **±** 10.0	216.3 **±** 51.8	0.042 **±** 0.014	551.4 **±** 153.7	1.94 **±** 0.2	4.4 **±** 0.4
UFCAPs	50.7 **±** 3.7	9.7 **±** 0.2	6.7 **±** 0.4	87.0 **±** 11.8	250.3 **±** 33.5	1.0E3 **±** 32.9	73.2 **±** 5.6	172.9 **±** 6.5	0.022 **±** 0.002	390.6 **±** 30.2	1.6 **±** 0.02	4.8 **±** 0.2
UFCAPs + O_3_	77.6 **±** 20.3	10.6 **±** 0.8	5.3 **±** 0.5	120.2 **±** 31.4	158.7 **±** 25.3	1.0E3 **±** 178.5	65.1 **±** 6.6	239.5 **±** 59.1	0.015 **±** 0.004*	441.8 **±** 61.4	2.0 **±** 0.2	4.7 **±** 0.2
FCAPs	58.5 **±** 13.7	9.8 **±** 0.5	6.0 **±** 0.1	94.9 **±** 14.3	191.3 **±** 23.3	1.2E3 **±** 124.2	86.9 **±** 12.9	190.1 **±** 32.0	0.023 **±** 0.005	396.9 **±** 71.5	1.8 **±** 0.1	5.0 **±** 0.2
FCAPs + O_3_	78.2 **±** 313.7	10.2 **±** 0.3	6.4 **±** 0.3	127.4 **±** 11.1	167.5 **±** 22.2	1.9E3 **±** 499.5	55.6 **±** 9.6	170.1 **±** 20.5	0.019 **±** 0.003	382.4 **±** 53.6	1.9 **±** 0.2	4.3 **±** 0.1
O_3_	53.9 **±** 7.5	9.5 **±** 0.4	5.0 **±** 0.2	83.7 **±** 6.3	142.4 **±** 30.3	1.7E3 **±** 334.0	71.9 **±** 18.0	203.9 **±** 36.4	0.016 **±** 0.004*	431.9 **±** 86.1	2.6 **±** 0.3	4.5 **±** 0.3

Values are mean ± SEM. *p < 0.05; significantly different from FA.

LDH – Lactate dehydrogenase.

MIA – Microalbumin.

NAG - N-acetyl-b-d-glucosaminidase.

ACE – angiotensin converting enzyme.

CK – Creatine Kinase.

CRP – C-reactive protein.

HBDH - α-hydroxybutyrate dehydrogenase.

GTR – glutathione-S-transferase.

SOD – Superoxide dismutase.

## Discussion

This study demonstrates that a single inhalation exposure to either FCAPs or UFCAPs differentially affects cardiac mechanical and electrical responses in mice, and that the effect of O_3_ co-exposure on the response varies for each particle size. FCAPs alone caused decreased ventricular contractility but contrary to our original hypothesis UFCAPs alone had no effect. However, introduction of O_3_ as a co-pollutant with UFCAPs caused a significant decrease in cardiac contractility 24 hours after exposure and blunted the effects of FCAPs. In contrast, although exposure to either FCAPs or UFCAPs alone did not cause any significant electrocardiogram effects, co-exposure to each with O_3_ caused electrical and HRV changes that might indicate increased cardiac risk. Overall, our results demonstrate that UFCAPs + O_3_ produces the most significant effects across both mechanical and electrical cardiac function (Table 5). Thus, these data suggest there is a differential effect of particle size, which holds true in the presence or absence of O_3_, confirming the health effects resulting from a PM-gas co-exposure are not simply the sum of both pollutants. Instead, it appears each interaction (FCAPs + O_3_ vs. UFCAPs + O_3_) is complex and needs to be examined separately, particularly when exposure concentrations are low and the responses are subtle.

**Table 5:** **Summary of effects**

**Group**	**LVDP**	**dP/dt**_**max**_	**dP/dt**_**min**_	**LVDP recovery (post-ischemia)**	**Coronary flow rate (post-ischemia)**	**Heart rate**	**SDNN**	**RMSSD**	**LF/HF**	**QTc**	**NCPW**
FA	--	--	--	--	--	--	--	--	--	--	--
UFCAPs	NE	NE	NE	NE	NE	NE	NE	NE	NE	NE	NE
UFCAPs + O_3_	↓	↓	↓	NE	NE	NE	NE	NE	NE	↑	↑
FCAPs	↓	↓	↓	NE	NE	NE	NE	NE	NE	NE	NE
FCAPs + O_3_	NE	NE	NE	NE	NE	NE	↓	NE	NE	NE	NE
O_3_	NE	NE	NE	NE	NE	NE	NE	NE	NE	NE	NE

NE = No Effect.

↓ = significant decrease.

↑ = significant increase.

NOTE: the above responses are compared to Air.

Our previous findings [21] suggested that UFCAPs would cause greater cardiac effects than FCAPs. Ultrafine black carbon particles have been shown to translocate into the blood circulation and have the potential to cause direct effects on the cardiovascular system [23,24]. UF particles cause heterogeneity of repolarization and decreased HRV in humans [25], whereas mechanical assessments in animals reveal decreased LVDP, contractility and coronary flow [20,21,26,27]. In this study, animals were exposed via whole-body inhalation as opposed to instillation [21,26], direct perfusion [20,27], or nasal inhalation [22], which could have resulted in a comparatively lower effective dose and milder response [28,29]. However, among our animals, calculations of estimated total dose indicated that there was no difference between UFCAPs and FCAPs suggesting that in the absence of O_3_ more than just particle burden was responsible for the cardiac decrements. Instead, the site of deposition (i.e. pulmonary vs. extra-thoracic) may have played a more important role. FCAPs, which we estimated had a higher extra-thoracic deposition when compared to UFCAPs (0.232 μg vs. 0.142 μg, respectively), may have caused its effects through the activation of upper airway sensory mechanisms. Previous studies have shown that PM_2.5_ can cause irritation and subsequent activation of autonomic reflex arcs, particularly due to the presence of acidic components; UF particles did not produce the same response [30]. Thus, the higher relative exposure concentrations and differential deposition of FCAPs may have resulted in variable epithelial injury, inflammation, clearance and thus toxicological presentation [31].

On the other hand, it is not entirely surprising that on their own FCAPs and UFCAPs did not cause any significant changes in ECG given our previous negative results with a more toxic pollutant [32]. Similarly, Campen et al. [33] found that Apolipoprotein E (ApoE) -/- mice on a high fat diet, which are assumed to be susceptible to the cardiotoxic effects of inhaled pollutants, did not have any ECG changes when exposed to high concentrations of road dust PM or the vapor phase of gasoline engine exhaust. As far as arrhythmias are concerned, spatial dispersion of cardiac repolarization, which contributes to arrhythmogenesis, is increased in people after co-exposure to CAPs and O_3_ with each pollutant causing minimal effects on their own [34]. Even in the presence of O_3_, it is clear from not only our results, but the previously mentioned human data and other humans studies [35], that relatively low CAPs exposures will likely only cause mild electrical and HRV changes in healthy populations. Thus, a significant ECG effect due to acute exposure may not necessarily be direct evidence of serious cardiovascular morbidity or premature mortality; rather, it may reflect a transient instability that can worsen if exposure continues over a longer period.

Co-exposure to UFCAPs and O_3_ produced electrophysiological changes indicative of increased heterogeneity of repolarization, as well as an increased incidence of non-conducted p-wave arrhythmias, which suggest atrioventricular block. In humans, this form of arrhythmia is usually seen with a wide QRS complex [36], which was also observed in our mice exposed to UFCAPs + O_3_; this sometimes indicates that conduction is impaired in the ventricles, particularly when observed with a block. These results corroborate findings from human studies of PM exposure [37,38] as well as human studies of PM and O_3_ co-exposure [34]. Similarly, a long QTc due to prolonged repolarization suggests increased risk of early after-depolarizations, which can trigger arrhythmias and potentially myocardial infarction when propagated. Indeed it is not unusual that electrical and mechanical dysfunction were both observed in mice exposed to UFCAPs + O_3_ given increased arrhythmogenesis has been shown to be associated with changes in myocardial stretch [39].

Consequently, clarifying the role of each pollutant in the health response is challenging. It has been suggested that PM may be the main driver of the cardiovascular response in some instances with O_3_ acting as a modifier. Brook et al. previously showed that PM and O_3_ together cause acute arterial vasoconstriction in healthy humans subjects, but so does PM alone [3,40]. However, we observed depression of mechanical function with O_3_ alone; although much of the current research is focused on PM as a cardiotoxicant, several studies have also noted the adverse cardiovascular effects of O_3_ inhalation; which include decreased HR, alteration of cardiac repolarization, and increased inflammation [9,41-43]. As such, the type of cardiac responses following air pollution may be dependent on the type of pollutant, or combination of pollutants, with some degree of overlapping effects. Tankersley et al. [44] showed that both carbon black particles and O_3_ caused reduced cardiac output in mice but due to two different mechanisms. Thus, we speculate that although both particles and gases produce similar cardiac decrements, the mechanisms mediating the response may not be the same (e.g. translocation vs. airway sensory irritation). Combinations of pollutants only complicates the assessment due the involvement of various separate or overlapping mechanisms. Regardless, the responses appear to be independent of total particle dose or even pulmonary deposition given UFCAPs was estimated to be less than FCAPs (pulmonary dose - 0.104 μg vs. 0.135 μg, respectively).

The role of the autonomic nervous system cannot be entirely discounted either; as demonstrated through HRV, the responses observed here and in other studies with respect to PM exposure appear to be dependent on the size of the particles. The interpretation and importance of HRV in air pollution studies is still not entirely agreed upon, particularly when examining populations with underlying cardiovascular disease. Mills et al. [45] and Peretz et al. [46] did not observe any HRV changes in humans exposed to diesel exhaust, however this lack of effect does not necessarily imply that there are no autonomic changes, instead a trigger (e.g. stress, exercise, etc) may be necessary to reveal any HRV differences. On the other hand, some studies show that particles, especially fine, cause HRV effects in humans. Several studies have demonstrated that exposure to FCAPs causes decreased HRV in young healthy or elderly adults [15,47,48] with O_3_ co-exposure only potentiating the response [15]. In healthy young adults, there was no dose-response relationship between FCAPs mass and HRV, however when combined with O_3_, increases in CAPs mass decreased HRV in a dose-dependent manner [35]. On the other hand, UFCAPs either have no effect [49] or increase HRV [17,25] or the results are less conclusive across all studies. Long-term exposure to UFCAPs, or a higher concentration, may have caused a significant change in HRV given these particles have the ability to penetrate deep into the lung, cause inflammation and activate autonomic reflex pathways [50].

Some of these pathways may lead to subsequent ischemic damage, which has been shown to be increased by PM. Cozzi et al. showed that in mice intra-tracheally instilled with ultrafine PM, infarct size and oxidative stress in the myocardium were significantly increased [51]. This corroborates our previous PM instillation studies which also demonstrated an increase in post-ischemia infarct size and decreased recovery of LVDP [21]. It appears that the method of exposure significantly impacts the post-ischemia response because even though exposure to FCAPs or UFCAPs + O_3_ caused significant pre-ischemia functional decrements, there was no change in coronary flow post-ischemia and there appeared to be an improvement of LVDP recovery (Figure 7). These findings are similar to what we observed with inhalation of multipollutant mixtures [43] and may represent activation of some compensatory mechanism post-exposure that actually protects the heart during ischemic injury. Lastly, although infarct size was not measured in our animals, we theorize that there was probably minimum to no increase particularly given we previously observed a decrease in infarct size with multipollutant mixture inhalation [43]. Thus, acute inhalation of fine or ultrafine PM alone or in combination with O_3_ may not be potent enough to cause serious ischemia-related damage and that a higher concentration is necessary to overcome this apparent response threshold.

Other than the mode of exposure, the chemical and physical characteristics of the PM might also account for some of the differences in response observed in this study. Indeed it is a limitation that exposure to CAPs alone could not be done on the same days as CAPs + O_3_; this accounts for the variation in not only particle numbers but composition as well. However, it is our assertion that the responses to these “real-world” particle concentrations are important, especially given the daily fluctuation of particulate air pollution and the ubiquitousness of O_3_. It is also important to note that although we compare these results to our previous study [21], the composition of the current FCAPs and UFCAPs is different. Our CAPs, particularly the UFCAPs, had a higher organic (OC) and total carbon (TC) content; thus possibly explaining the differences in response.

As such, there was a three-fold decrease in Cu and a two-fold decrease in Fe in the FCAPs + O_3_ exposure when compared to FCAPs alone which may have contributed to the lack of effect in the former. There was also an increase in Ni and Cu, which have been shown to be two of the most toxic metals found in PM [52], in the UFCAPs + O_3_ exposure when compared to UFCAPs alone. In contrast, even though it appears that mass was not a factor in the observed decrements because there was less PM in the UFCAPs + O_3_ exposure than UFCAPs alone and the opposite for the FCAPs and O_3_ co-exposures, there was a significantly higher sulfate and OC/TC content in the UFCAPs, especially the UFCAPs + O_3_, when compared to FCAPs, which may explain the larger cardiac effect [53]. On the other hand, responses to FCAPs and UFCAPs combined with O_3_ might also be partially explained by the chemical changes occurring in PM upon ozonization. Ozone is highly reactive and therefore it has the potential to react with certain components of PM such as the aromatic compounds [6]. It has been documented that ozonization of aromatic substances can result in the formation of carbonyls, carboxylic acids, quinones, and epoxides, which can be more toxic than the parent compound [54,55], but also less potent due to “over-ozonization” [56]. It is yet unclear which mechanism is at play here.

Additionally, O_3_ may cause epithelial injury and oxidative stress, which facilitate the PM effects [57]. Adamson et al. [58] showed that O_3_ and urban particulate co-exposure resulted in greater epithelial injury and interstitial inflammation than for either component alone; not to mention UFP did not have a large biological effect without O_3_. As such, co-exposures may produce differential responses due to toxicological interactions within the host. Thomson et al. [59] showed that on their own, PM and O_3_ increased expression of the potent vasoconstrictor endothelin-1 (ET-1) in the lungs and its circulating levels in the plasma, however, together they only caused an upregulation (i.e. without plasma “spill-over”). Although there were no significant changes in inflammatory cells or markers in the blood or lavage, we found that O_3_ alone and UFCAPs + O_3_, but not FCAPs or UFCAPs alone, caused significantly decreased serum glutathione S-transferase (GTR) levels, which is indicative of increased oxidative stress; direct measurement of oxidative stress in the myocardium may have revealed a greater involvement as was shown by Cozzi et al. [51]. Wang et al. previously showed that PM_2.5_ and O_3_ increased several markers of inflammation and oxidative stress in rats however their exposure concentrations were significantly higher than those used here [60]. Regardless, synergistic interactions between inhalable PM and O_3_ can increase the generation of reactive oxygen species due to the porous surface of particles which provides ample surface area for reactivity, but that the potency still depends on particle concentration, size and other factors [61,62].

## Conclusion

The results of this study demonstrate that fine and ultrafine CAPs differentially alter cardiac responses, which include both mechanical and electrical effects. More importantly, these data clearly show that the effects of co-exposure may not be simply additive or synergistic, nor even generalizable. Although only fine CAPs had significant effects on its own, O_3_ co-exposure with FCAPs caused decreased HRV whereas with UFCAPs caused electrical changes and arrhythmia. Interestingly, O_3_ co-exposure only caused mechanical decrements with UFCAPs and to our surprise blunted the effects of FCAPs. This indicates that the size, and potentially the chemical composition, of the particle is an important determinant of the type of cardiac response, particularly when gaseous co-pollutants are present. Although the responses were subtle, the important message may be that latent underlying changes are occurring post-exposure and that the deleterious effects of even a single exposure to air pollution needs to be considered. Some of these might not manifest as overt symptoms, however the latent effect might not be any less serious, instead increasing the susceptibility to subsequent triggered adverse responses (i.e. due to loss of compensatory capacity), particularly in people with existing cardiovascular disease.

## Materials and methods

### Animals

Ten to twelve-week old female C57BL/6 mice (body weight = 21.6 ± 0.1 g) were used in this study (Jackson Laboratory - Bar Harbor, ME). Mice were initially housed five per cage and maintained on a 12-hr light/dark cycle at approximately 22°C and 50% relative humidity in an AAALAC–approved facility. Food (Prolab RMH 3000; PMI Nutrition International, St. Louis, MO) and water were provided ad libitum. Each mouse implanted with a radiotelemeter was singly housed after surgery. All protocols were approved by the Institutional Animal Care and Use Committee of the U.S. Environmental Protection Agency and are in accordance with the National Institutes of Health Guides for the Care and Use of Laboratory Animals. The animals were treated humanely and with regard for alleviation of suffering.

### Experimental groups

Mice were randomly assigned to one of six exposure groups: (1) fine concentrated ambient particles (FCAPs); (2) ultrafine CAPs (UFCAPs); (3) ozone (O_3_); (4) FCAPs and O_3_ co-exposure (FCAPs + O_3_); (5) UFCAPs and O_3_ co-exposure (UFCAPs + O_3_); and (6) filtered air (FA). Each group had n = 6. Separate groups (same as above) of mice were used for Langendorff cardiac perfusion experiments (n = 5-8).

### Surgical implantation of radiotelemeters

Animals were weighed and then anesthetized using inhaled isoflurane (Isothesia, Butler Animal Health Supply, Dublin OH). Anesthesia was induced by spontaneous breathing of 2.5% isoflurane in pure oxygen at a flow rate of 1 L/min and then maintained by 1.5% isoflurane in pure oxygen at a flow rate of 0.5 L/min; all animals received the analgesic Buprenorphrine (0.03 mg/kg, i.p. manufacturer). Briefly, using aseptic technique, each animal was implanted subcutaneously with a radiotelemeter (ETA-F10, Data Sciences International, St Paul, MN); the transmitter was placed under the skin to the right of the midline on the dorsal side. The two electrode leads were then tunneled subcutaneously across the lateral dorsal sides; the distal portions were fixed in positions that approximated those of the lead II of a standard electrocardiogram (ECG). Body heat was maintained both during and immediately after the surgery. Animals were given food and water post-surgery and were housed individually. All animals were allowed 7-10 days to recover from the surgery and reestablish circadian rhythms.

### Radiotelemetry data acquisition

Radiotelemetry methodology (Data Sciences International, Inc., St. Paul, MN) was used to track changes in cardiovascular function by monitoring heart rate (HR), and ECG waveforms immediately following telemeter implantation, through exposure until 24 hours post-exposure. This methodology provided continuous monitoring and collection of physiologic data from individual mice to a remote receiver. Sixty-second ECG segments were recorded every 5 minutes during the pre- and post-exposure periods and continuously during exposure (baseline and hours 1-4); HR was automatically obtained from the waveforms (Dataquest ART Software, version 3.01, Data Sciences International, St. Paul, MN, USA).

### Electrocardiogram analysis

ECGAuto software (EMKA Technologies USA, Falls Church VA) was used to visualize individual ECG waveforms, analyze and quantify ECG segment durations and areas, as well as identify cardiac arrhythmias as previously described [63]. Briefly, using ECGAuto, P-wave, QRS complex, and T-wave were identified for individual ECG waveforms and compiled into a library. Analysis of all experimental ECG waveforms was then based on established libraries. The following parameters were determined for each ECG waveform: PR interval (P_start_-R), QRS complex duration (Q_start_-S), ST segment interval (S-T_end_) and QT interval (Q_start_-T_end_). QT interval was corrected for HR using the correction formula for mice QTc = QT/(RR/100)^1/2^ [64]. Figure 3A and B show a typical ECG trace as well as a typical non-conducted p-wave (NCPW) arrhythmia, which indicates an intermittent atrioventricular block, as observed in mice, respectively.

### HRV analysis

Heart rate variability (HRV) was calculated as the mean of the differences between sequential RRs for the complete set of ECG waveforms using ECGAuto. For each 1-min stream of ECG waveforms, mean time between successive QRS complex peaks (RR interval), mean HR, and mean HRV-analysis–generated time-domain measures were acquired. The time-domain measures included standard deviation of the time between normal-to-normal beats (SDNN), and root mean squared of successive differences (RMSSD). HRV analysis was also conducted in the frequency domain using a fast-Fourier transform. The spectral power obtained from this transformation represents the total harmonic variability for the frequency range being analyzed. In this study, the spectrum was divided into low-frequency (LF) and high-frequency (HF) regions. The ratio of these two frequency domains (LF/HF) provides an estimate of the relative balance between sympathetic (LF) and vagal (HF) activity.

### Concentrated ambient particle and ozone exposure

See Additional file 1 for full exposure details. Briefly, concentrated ambient particles (CAPs) and ozone (O_3_) were generated in the U.S. EPA’s Concentrated Air Particles Laboratory, Research Triangle Park, NC. All exposures were carried out in the summer months of June and July and under sunny and warm climate conditions. Ambient air containing PM from outside the facility entered the systems and passed through a size selective inlet removing PM > 2.5 μm so that remaining particles were in the size fractions of interest. The largest source of PM was from mobile sources (≈20%), wood combustion (≈21%), road dust (≈4%) and other minor sources such as brake wear and marine salt; the remaining PM was from secondary sulfates (≈50-55%).

Incoming air was then split into two streams and particles were selectively concentrated into either the fine (0.1 to 2.5 μm) or ultrafine mode (<300 nm) and then delivered into two separate chambers. Real time measurements of number concentration and particle size distribution were performed using a scanning mobility particle sizer (SMPS) and an Aerodynamic Particle Sizer (APS). A generator was used to produce O_3_ (0.3 ppm), which was then delivered to a third chamber. Chamber plumbing was altered to allow different configurations of concentrated PM and/or O_3_ including: FCAPs alone, UFCAPs alone, FCAPs + O_3_, UFCAPs + O_3_, O_3_ alone, or filtered air (FA). Exposure to FCAPs/UFCAPs alone had to be done on separate days from FCAPs/UFCAPs co-exposures with O_3_ due to limitations in the exposure system (i.e. exposure to CAPs alone and CAPs + O_3_ could not be done on the same day); day-to-day variations in particle concentrations and composition were expected due to this.

The study protocol included two days of animal-to-chamber acclimatization prior to exposure. A normal four-hour exposure (Exp1 (exposure hour 1), Exp2, Exp3, and Exp4) started with one hour of additional chamber acclimatization (Baseline). All mice were moved back to their home-cages after the exposure (Recovery). The Multiple Pathway Particle Dosimetry (MPPD; Version 3.0) model was used to predict particle doses [65,66] for mice and humans; ventilatory parameters were estimated using typical values [67].

### Cardiac perfusion

The procedure for cardiac perfusion has been previously described [21]. Briefly, 24 hours after exposure, mice were anesthetized with sodium pentobarbital (80 mg/kg, i.p.). Heparin (100 units) was injected intravenously before removal of heart. The hearts were rapidly removed and placed in ice-cold Krebs-Henseleit buffer, after which the aortas were cannulated. Retrograde perfusion via the aorta was performed under constant pressure (100 cmH_2_O) above the heart. The non-recirculating perfusate was a Krebs-Henseleit buffer containing (in mmol/L) 120 NaCl, 5.9 KCl, 1.2 MgSO_4_, 1.75 CaCl_2_, 25 NaHCO_3_, and 11 glucose. The buffer was aerated with 95% O_2_—5% CO_2_ and maintained at pH 7.4 and a temperature of 37°C.

For assessment of contractile function, a latex balloon on the tip of a polyethylene catheter was inserted through the left atrium into the left ventricle. The catheter was connected to a pressure transducer (Argon Medical Devices, Athens, TX) at the same height as the heart. The pressure of the left ventricular balloon was inflated to 0-5 cmH_2_O. A PowerLab system was used to collect and process the heart rate, left ventricular developed pressure (LVDP = LV peak minus end-diastolic pressure (LVEDP)), and contractility (dP/dt) data (AD Instruments, Milford, MA). All hearts were perfused for 25 min; we then initiated 20 min of global no-flow ischemia by stopping the flow of oxygenated perfusion buffer, followed by 1 h of reperfusion. Onset of ischemic contracture was measured as the time from the start of ischemia until initial contracture (at least 5 cmH_2_O increase in left ventricular pressure). Recovery of LVDP, expressed as a percentage of the initial pre-ischemic LVDP, was measured at 20, 40 and 60 min of reperfusion after 20 min of ischemia.

### Tissue collection and analysis

See Additional file 1 for full details, procedures were performed as previously described [32]. Briefly, 24 hrs after exposure, mice were euthanized and blood and lung lavage fluid (BAL) were collected, processed and analyzed. Multiple biochemical markers (e.g. lactate dehydrogenase, protein, etc) were assessed in the BAL, and serum or plasma supernatants were analyzed for creatine kinase, C-reactive protein (CRP), and other markers to assess cardiopulmonary inflammation, injury and oxidative stress.

### Statistics

All data are expressed as means ± SEM. Statistical analyses of the data were performed with GraphPad Prism 5 (GraphPad software, San Diego CA). For HR, ECG intervals and HRV, two-way analysis of variance (ANOVA) for repeated-measures and Bonferroni *post hoc* tests were used to determine statistical differences. A one-way ANOVA was used to analyze arrhythmia counts. For Langendorff cardiac perfusion data, comparisons between groups were performed by one-way ANOVA followed by Bonferroni post hoc test for multiple comparisons. Comparisons were made across all groups taking into account the multiple endpoints, exposure groups and time points as well as any interactions. An oblique principal component cluster analysis and multivariate analysis of variance (MANOVA – GLM procedure and least squares means post hoc test) were performed using SAS version 9.3 software, (SAS Institute Inc, Cary, NC) to determine whether the elements found in the CAPs on their own or in combination with one another had an effect on the cardiac responses. The objective of this approach was to reduce the large number of variables (i.e. elements) to a smaller set that still retain the information in the original data set and then examine for effects. Five clusters were revealed and elements belonging to the same cluster had strong correlations. A p-value < 0.05 was considered statistically significant.

## Additional file

Additional file 1: **Supplementary Material.**

## Abbreviations

ACE: Angiotensin converting enzyme
BAL: Bronchoalveolar lavage
CRP: c-reactive protein
BP: Blood pressure
BRS: Baroreflex sensitivity
DEP: Diesel exhaust particles
dP/dt: 1st derivative of the change in left ventricular pressure/time
ECG: Electrocardiogram
EC: Elemental carbon
ET-1: Endothelin-1
FA: Filtered air
FCAPs: Fine concentrated ambient particles
FMD: Flow-mediated dilation
GTR: Glutathione S-transferase
HF: High frequency
HR: Heart rate
HRV: Heart rate variability
I/R: Ischemia/reperfusion
LVDP: Left ventricular developed pressure
MAP: Mean arterial pressure
MDA: Malondialdehyde
NAG: N-acetyl-b-d-glucosaminidase
NCPW: Non-conducted p-wave
OC: Organic carbon
O_3_: Ozone
PM: Particulate matter
RMSSD: Root mean square of successive differences of NNs
SDNN: Standard deviation of NN intervals
SOD: Superoxide dismutase
T_co_: Core body temperature
TC: Total carbon
TRPA1: Transient receptor potential cation channel, member A1
UFCAPs: Ultrafine concentrated ambient particles
