# Supplementary material for: Ozone co-exposure modifies cardiac responses to fine and ultrafine ambient particulate matter in mice: concordance of electrocardiogram and mechanical responses
Source: Part Fibre Toxicol. 2014 Oct 16;11:54. doi: 10.1186/s12989-014-0054-4 (PMC4203862; doi:10.1186/s12989-014-0054-4)
Supplement: Supplementary file 1 — Supplementary Material. [file 12989_2014_54_MOESM1_ESM.docx]

**Additional file 1**

**Ozone co-exposure modifies cardiac responses to fine and ultrafine ambient**

**particulate matter in mice: concordance of electrocardiogram and mechanical responses**

Nicole Kurhanewicz^1*^, Rachel McIntosh-Kastrinsky^2*^, Haiyan Tong^3^, Leon Walsh^3^, Aimen Farraj^3^ and Mehdi Hazari^3^

**MATERIALS AND METHODS**

*Concentrated ambient particle and ozone exposure* **-** Concentrated ambient particles (CAPs) and ozone (O_3_) were generated in the U.S. EPA’s Concentrated Air Particles Laboratory, Research Triangle Park, NC. Fine (FCAPs) and ultrafine (UFCAPs) CAPs are defined as having an aerodynamic diameter of 0.1 to 2.5 µm and 100 nm and below, respectively. Ambient air (10,000 L/m) containing PM from outside the facility entered the systems, was transported via heated stainless steel ductwork, and passed through a size selective inlet removing PM > 2.5 μm so that remaining particles were in the size fractions of interest. Air was split into two 5000 L/m streams and delivered to specialized particle concentrating equipment and then separate chambers for animal exposures: (1) 5000 L/m stream was delivered to a Harvard Fine Mode Concentrated Air Particles system, which concentrated fine mode particles between 0.1 and 2.5 μm using three stages of slit virtual impactors producing an output of approximately 50 L/m air with PM concentrated up to 30 times the entry levels depending on particle size; and (2) 5,000 L/m stream passed thru a Harvard Ultrafine Concentrated Air Particles system, which concentrated ultrafine PM of < 300 nm into a 58 L/m flow. An inline size selective outlet removed PM greater than ~300 nm from the air stream prior to delivery to the UFCAPs exposure chamber. Output concentration of each system was dependent on the makeup and condition of the entering ambient PM fractions and varied over time with changing ambient conditions. Exposures were carried out in three stainless steel and glass whole-body exposure chambers with adjustable inlet plumbing and pollutant gas injection allowing multiple exposure configurations.

Ozone was generated by passing extra dry oxygen past an arcing transformer in a model V5-0 O_3_ generator (Ozone Research & Equipment Corp., Phoenix, AZ). The chamber concentration (0.3 ppm) was controlled by the computer program DASYLab (version 9.0; DasyTec USA, Amherst, NH), which controlled the opening and closing of a mass flow controller at each chamber. The actual concentration was then read by an O_3_ analyzer (model 400; Teledyne-Advanced Pollution Instruments, Inc., Thousand Oaks, CA), which fed a signal to a proportional, integral, derivative loop control, which then either opened or closed the mass flow controller to maintain the O_3_ concentration in the chamber at the desired level.

Chamber plumbing was altered to allow different configurations of concentrated PM and/or O_3_ including: FCAPs alone, UFCAPs alone, FCAPs+O_3_, UFCAPs+O_3_, O_3_ alone, or filtered air (FA). The study protocol included two days of animal-to-chamber acclimatization prior to exposure. A normal exposure started with one hour of additional chamber acclimatization. All mice were moved back to their home-cages after the exposure. Chamber environment and system operating parameters were continuously monitored, controlled, and recorded using computerized data acquisition and control systems (DAS) (DASYLab Pro, v9.0, Data Acquisition System Laboratory, Measurement Computing Corp.). Particle size and number concentration was continuously monitored and recorded using TSI scanning mobility particle sizer (SMPS) and APS II particle instruments. Exposure conditions such as temperature, relative humidity, O_3_ concentration, PM mass concentration (µg/m^3^), number concentration (pt/cc), and particle size (nm) were averaged for all animals in each exposure group. PM samples were individually analyzed and the data for all samples of each group were then averaged to establish the group’s results.

*Tissue collection and analysis.* Twenty-four hours after exposure, mice were deeply anesthetized with an intra-peritoneal injection of a sodium pentobarbital/phenytoin solution. Blood and lung lavage fluid (BAL) were collected, processed, and analyzed as previously described (Hazari et al. 2009). To assess cardiopulmonary inflammation, injury and oxidative stress, multiple biochemical markers and inflammatory cells were assayed. Lavage supernatants were analyzed for lactate dehydrogenase (LDH), N-acetyl-b-d-glucosaminidase (NAG), microalbumin (MIA), and total protein. Serum supernatants were analyzed for creatine kinase (CK), C-reactive protein (CRP), total protein, and glutathione-S-transferase (GTR); and supernatants from plasma were assayed for angiotensin converting enzyme (ACE). Serum was also analyzed for α-hydroxybutyrate dehydrogenase (HBDH), lactate dehydrogenase-1 (LDH), superoxide dismutase (SOD) according to previous procedures (Farraj et al. 2009).
